# Normal axillary thickness thresholds as a metric for nutritional status of children^☆^

**DOI:** 10.1016/j.clinimag.2018.11.010

**Published:** 2019

**Authors:** Alissa L. Wall Kleinhenz, Jing Gao, Arzu Kovanlikaya, Daniel Rosenbaum, Daniela I. Guisado, Jonathan M. Rubin

**Affiliations:** a University of Michigan Medical School, Taubman Health Sciences Library, 1135 Catherine St, Ann Arbor, MI 48109, United States of America; b Rocky Vista University, RVUCOM-SU Campus Faculty Suites, 255 E. Center Street, Ivins, UT 84738, United States of America; c Department of Radiology, Weill Cornell Medical School, 525 East 68th Street, New York, NY 10065, United States of America; d Department of Radiology, BC Children's Hospital, 4480 Oak Street, Vancouver, BC V6H 3V4, Canada; e Weill Cornell Medical School, New York, NY, United States of America; f Department of Radiology, University of Michigan Medical School, 3208C Medical Sciences Building 1, B1D502, Ann Arbor, MI 48109-5030, United States of America

**Keywords:** Lung ultrasound, Childhood pneumonia, Nutritional metrics, Axillary CT scans

## Abstract

**Introduction:**

Childhood pneumonia is a major cause of death in the 3rd world, and undernourishment increases the severity of the condition. We considered axillary thickness as a simple measurement to evaluate nutritional status that can be performed simultaneously with lung ultrasound. Our goal was to determine the distribution of axillary thickness in a cohort of children to determine a threshold for malnutrition.

**Methods:**

Clinical databases were scanned to identify chest computed tomograms (CT) in children between the ages of 0 and 5 years with non-debilitating disease. The bilateral axillary thicknesses of the cohort were determined using equivalent width, and these measurements were segmented by age, sex, and laterality to determine cutoff thresholds. Data was evaluated using single factor analysis of variance (ANOVA) and 5th percentile analysis to determine the lower bound thresholds of axillary thickness.

**Results:**

247 scans met inclusion criteria. ANOVA demonstrated no significant differences in the mean measurements in the 5 groups (*p* = 0.377). 95% confidence limits on the 5th percentile plots showed an axillary thickness of 1.5 cm was a reasonable threshold for malnutrition detection for all age groups and sexes except for males between 0 and 1 years old where a 1.1 cm threshold may be required.

**Discussion:**

CT scans of the chests in a cohort of children without debilitating disease revealed a remarkably uniform axillary thickness threshold for malnutrition assessment of 1.5 cm. This suggests that there may be a threshold for nutritional assessment for children undergoing lung ultrasound scans for childhood pneumonia.

## Highlights

• Determined normal axillary thickness distributions in children aged 0–5 as a proxy to reflect childhood nutritional status
• Equivalent width of axillae were determined from CT scans as a measure of thickness
• A cutoff of 1.5 cm suffices for all ages except for boys between 0 and 1 years of age where 1.1 cm may be more appropriate

## 1 Introduction

Evaluating the nutritional state of a child provides critical diagnostic and prognostic information about a child's health, and can influence a multitude of pathologic processes including childhood pneumonia. Undernourishment magnifies the risk of developing pneumonia, and contributes to >1 million deaths in the 0–4 year age range [1]. In the pathogenesis of pneumonia, decreased protein and energy reserves lead to a blunted immune response and weakened respiratory muscles, thereby limiting a child's ability to clear respiratory secretions [1,2]. Hence, it is important to identify undernourished children and intervene to reverse the condition before a child becomes ill, such as by breast feeding or augmenting zinc intake [[3], [4], [5], [6]]. Given the magnitude of the medical problem represented by childhood pneumonia and associated malnutrition, improved diagnosis and assessment of these conditions is a high priority.

Cellphone technology is rapidly spreading throughout the world, and a large fraction of the world's population now has access to cellphones service [7]. New technologies have converted cellphones into ultrasound scanner displays by simply attaching a transducer with beamforming capabilities in its handle [8]. New capacitive micromachined ultrasound transducers (CMUT) in complementary metal-oxide-semiconductor (CMOS) techniques should make it possible to produce ultrasound scanners on a chip that will only cost pennies to fabricate [9].

Presently, ultrasound scans of the lungs and chest are one of the fastest growing areas of ultrasound imaging with diagnostic capabilities far beyond the classic application of pleural effusion localization [[10], [11], [12], [13]]. Ultrasound imaging is now being used to assess tumors, pulmonary edema, and pneumonia [10,12,13]. Further, ultrasound strain imaging has been shown in preliminary studies to be able to assess local lung ventilation in real-time [14]. A reasonable question to ask is: could a child's nutritional state be estimated using ultrasound imaging simultaneously with lung ultrasound?

There are many physical metrics that have been employed to estimate nutritional status of children. These include body mass indices, weight vs height/length metrics, skin fold thicknesses of soft tissues overlying the triceps or subscapularis muscles, head circumference measurements among others [15,16]. Another possible similar metric of nutritional state could be axillary thickness. The axillae typically contain fat, so an undernourished child's axillae would reasonably be considered to contain less than the normal quantity of fatty tissue. The thickness of the axillae, as a metric of the amount of fatty tissue, could easily be measured during ultrasound imaging for lung motion. Thus, a simple digital binary display could inform the examiner if the child were undernourished or not—making this streamlined approach advantageous over current measurements of malnutrition such as skinfold thickness. However, in order to make this assessment, the distribution of the normal thicknesses of the axillae must be known. It is with this goal in mind that we undertook this study in which we use CT scans of the chest in a population of children without chronic disease to define the axillary measurement thresholds for differentiating between normally nourished and malnourished populations. Previous studies in other anatomic regions have demonstrated high concordance between CT and ultrasound measurements [[17], [18], [19]]. Thus, we sought to determine normal axillary thickness distributions in children ages 0–5 years old.

## 2 Methods

This retrospective study was approved by the institution review boards of both participating institutions. No informed consents were obtained.

The goal of this study is to determine normal axillary thickness distributions in children ages 0–5 years old. The ideal population would be patients with no evidence of disease who had screening chest computed tomograms (CT) for detecting occult disease in the chest. However, screening scans are not performed on children. Thus, to identify a normally nourished population with chest CT scans, we identified axial CT scans of the chest in children without evidence of chronic disease that could be associated with weight loss or decreased growth. Such diseases would include scans to identify vascular malformations or vascular rings, acute scans for asthma without prior history of pneumonia, scans to rule out acute pneumonias, or acutely identified masses in the abdomen that have not been biopsied or treated. Patients with evidence of chronic disease through history, prior CT scans for the same condition, prior surgery or other treatment of the same condition were excluded. We, therefore, tried to identify patients most likely to be normally nourished by only including those with new-onset or acute indications for imaging. Databases from both participating academic institutions were scanned, one between the years of 1999–2016 and the other between 2011 and 2017, to identify the analyzed cases. Table 1 shows the distribution of conditions for which CT scans were performed.

**Table 1:** Distribution of clinical cases.

Indication	Total cases
Tumor staging/initial diagnosis	107
Vascular abnormality	49
Trauma	21
Structural lung abnormality	22
Pulmonary infection	29
Congenital abnormality	5
Interstitial lung disease	2
Infection	2
Congenital diaphragmatic hernia	3
Respiratory failure	2
Cyanotic episodes	1
Foreign body aspiration	1
Acute life threatening event	1
Myasthenia gravis	1
Preoperative evaluation	1
Total	247

**Fig. 1:**
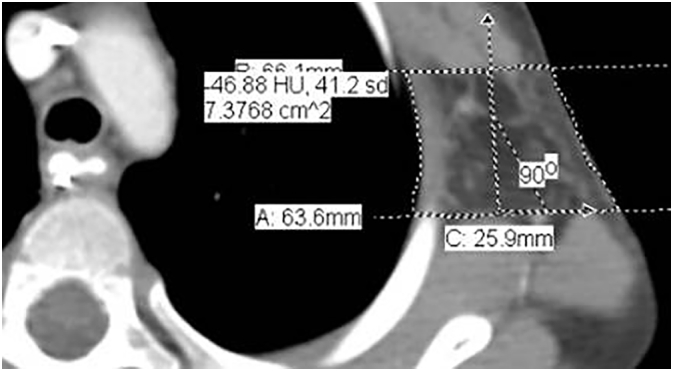
Cropped CT scan showing the left axilla with an equivalent width measurement. Two parallel lines define the margins of the pectoralis major and minor and the latissimus dorsi and subscapularis muscles. The lung surface and skin surface are defined with dotted lines connecting the two parallel lines. The equivalent width is 7.38 cm^2^/2.59 cm = 2.84 cm for the axillary depth. The line labeled 90° represents the normal between the two parallel lines along which the distance between the parallel lines is measured.

**Fig. 2:**
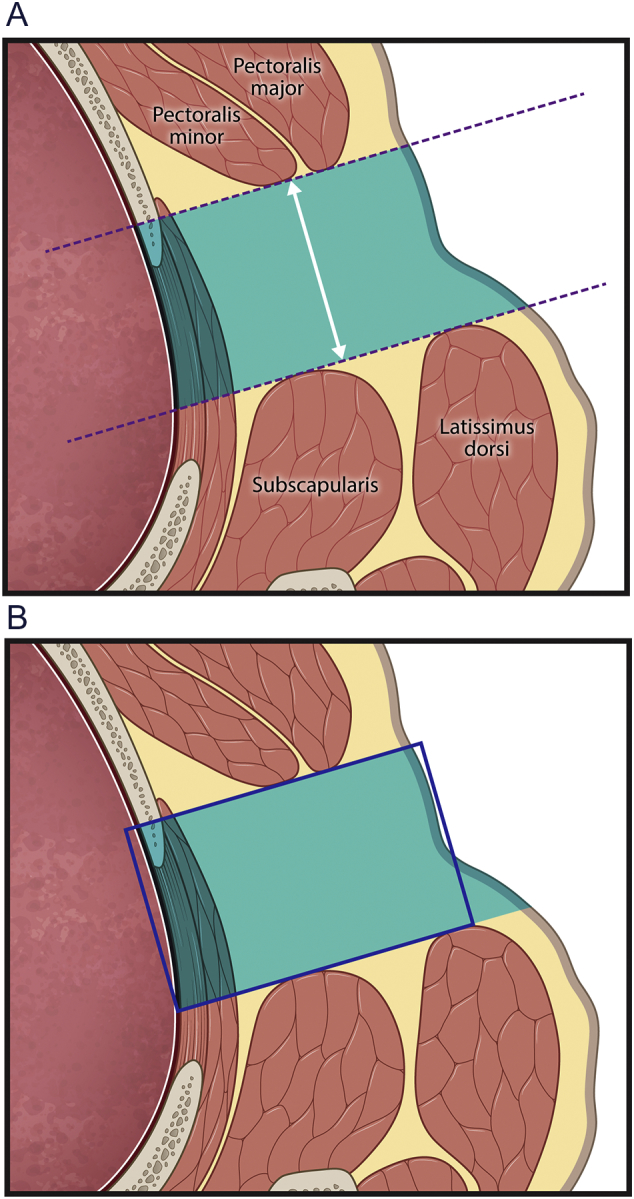
a) Line drawing of the anatomy of the left axilla used to define the equivalent rectangle. The muscle boundaries of the pectoralis major and minor anteriorly and the latissimus dorsi and subscapularis posteriorly define the two dotted parallel lines defining the axilla. The area encompassed by the two parallel lines, the skin surface, and the lung boundary is shown in blue, and the distance between the two parallel lines is depicted by the two headed arrow. b) The equivalent width rectangle with margins in dark blue is shown overlaying the axilla. The thickness of the axilla is defined as the side of the rectangle stretching between the lung and skin whose length is the area of the blue region in panel a divided by the length of the two headed arrow. (For interpretation of the references to color in this figure legend, the reader is referred to the web version of this article.)

We then measured axillary thickness bilaterally using the following protocol: We defined the axilla as the space between the pectoralis major/minor muscles anteriorly and the latissimus dorsi/subscapularis muscles posteriorly. Measurements were made at the level of the 2nd and 3rd rib interspace (Fig. 1, Fig. 2). A pair of parallel lines was drawn tangential to these two muscle groups. The perpendicular distance between the muscle groups was determined using each Radiology Department's CT display software.

In this construction, the region lying between these two parallel lines contains a segment of the skin surface at the lateral margin of each axilla and a segment of the lung surface at the medial boundary of each axilla. Using the skin surface as one boundary, the lung margin as another boundary, and the two parallel lines as third and fourth boundaries, we can define an enclosed region bounded by the skin, the lung, and the two parallel lines. The area of this region was estimated by drawing a simple, closed curve traced along the perimeter of this enclosed region. Dividing this area by the distance between the parallel lines gives a length measurement, i.e. the equivalent width, that represents the thickness of the axilla (Fig. 2). The equivalent width corresponds to the side of the rectangle that has the same area as the enclosed area defined above with one of the dimensions corresponding to the distance between the two parallel lines defining the margins of the pectoral muscles and the margin of the latissimus dorsi/subscapularis muscle. The equivalent width gives an axillary thickness measurement that compensates for the curved or irregular skin boundary at the superficial, lateral border of the axilla and the curved lung boundary at the medial border of the axilla, and we applied the equivalent width measurement as representative of the axillary thickness.

Because these CT scans were not performed to facilitate axillary measurements but rather to examine unrelated pathology, we limited the analysis to those axillae that could be measured using our equivalent width assessment method. Many scans were performed with one axilla excluded, or one or both arms positioned in a manner prohibiting our equivalent width method. Because of these caveats, we excluded scans of subjects where we could not make equivalent width calculations. First, if a subject's arms were down during scanning, the skin surface on the CT image was not well defined, prohibiting measurement. Second, sometimes the skin line was cut off on the image; in these cases, the axillary boundaries were not fully captured and could not be measured. Each of these contingencies may only affect one side or the other: for instance, one arm might be up while the other is down. This would mean that measurements could be made on one side only. However, given these caveats, we decided only to include subjects for which we could measure the axillary thickness on both sides. This further allowed us to test the intra-subject variation in the axillary measurements in the entire analyzed population (see below).

We separated the measurements into 5 cohorts: 0–1 years old, 1–2 years old, 2–3 years old, 3–4 years old, and 4–5 years old. We compared the groups' equivalent width measurements within each group and among the groups. We also segmented the groups within each cohort by sex and side (“left” versus “right”). Axillary thickness distributions for each group were analyzed for normality using the Kolmogorov-Smirnov test. After determining normality, measurements within and among the groups were compared using single-factor analysis of variance (ANOVA) to see if there were differences in the means of the groups. Cross-correlation analysis was performed to determine side differences in the measurements. *p* values < 0.05 were used to exclude the null hypothesis that there were no differences among the groups. 95% confidence limits were performed to determine if there were side differences in the regression analysis. These calculations were either performed in Excel (Microsoft Corporation, Redman, WA) or Python (Python Software Foundation, United States).

Further, since our aim in this study was to identify lower bounds of the axillary thickness distributions to determine thresholds for detecting malnutrition in children, we performed 5th percentile analyses on the thickness distributions. The distributions were plotted and the 5th percentiles with 95% confidence limits for these thresholds calculated using MatLab (Math Works, Andover MA). The 95% confidence intervals were calculated using a bootstrapping technique.

In order to test inter-observer variability, a random subset of 41 subjects was measured by two observers (AWK, JMR). A Bland-Altman analysis was performed to assess inter-observer variations.

## 3 Results

**Fig. 3:**
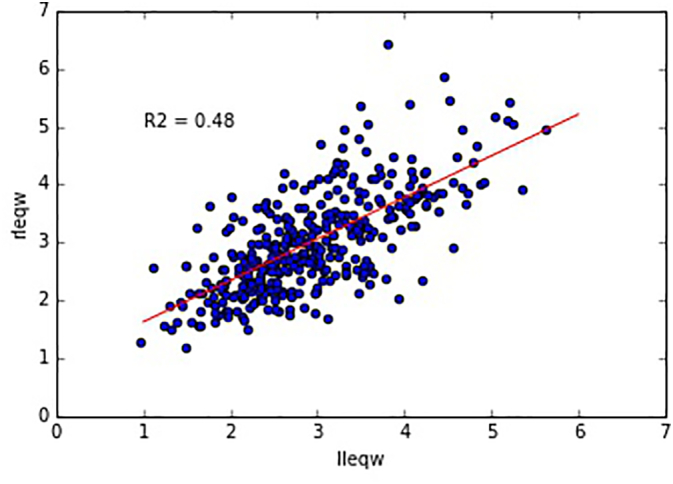
Scatter plot of the paired axillary data. The measurements are in centimeters. The regression equation is y = 0.716 ∗ x + 0.918. The 95% confidence intervals of the slope are (0.623, 0.828). These intervals do not intersect zero. rleqw = right lung equivalent width, lleqw = left lung equivalent width.

We included 247 children in the study. Table 1 shows the indications for the CT scans. These were further divided according to year cohorts and sex, and separated into right axillary and left axillary measurements (Table 2). Each of the cohorts was normally distributed based on Kolmogorov-Smirnov test (*p* > 0.05) (Table 2). A single factor ANOVA of the data shows no significant differences of the populations measurements over the 5 age ranges (*p* = 0.377). Correlation plot shows a significant trend in the means of the axillary thickness with a left side dominance over the population, i.e. the 95% confidence intervals of the slope do not include zero (Fig. 3).

**Table 2:** Table showing the distribution of the measurements in the 5 cohorts segmented by age, sex, and left and right sides. leqw= average of left and right lung equivalent widths for each case, std = standard deviation, kstest = Kolmogorov-Smirnov test statistic, rleqw = right lung equivalent width, lleqw = left lung equivalent width, n = number of cases in each category, F = female, M = male

		leqw				rleqw				lleqw			
		Mean (cm)	std (cm)	n	kstest	Mean (cm)	std (cm)	n	kstest	Mean (cm)	std (cm)	n	kstest
Age range	Sex												
0–1	F	2.815	0.744	37	0.198	2.875	0.842	37	0.617	2.754	0.712	37	0.283
0–1	M	2.949	0.807	40	0.984	3.017	0.816	40	0.702	2.881	0.882	40	0.997
1–2	F	2.752	0.623	24	0.541	2.879	0.757	24	0.892	2.625	0.638	24	0.508
1–2	M	3.250	0.807	29	0.575	3.278	0.789	29	0.734	3.222	0.963	29	0.817
2–3	F	2.604	0.545	18	0.998	2.636	0.574	18	0.742	2.572	0.574	18	0.894
2–3	M	3.012	0.762	15	0.703	3.038	1.113	15	0.136	2.987	0.797	15	0.972
3–4	F	2.911	0.804	19	0.430	2.970	0.864	19	0.766	2.851	0.890	19	0.723
3–4	M	3.213	0.671	25	0.776	3.292	0.777	25	0.594	3.134	0.649	25	0.653
4–5	F	2.746	0.479	11	0.916	2.820	0.666	11	1.000	2.672	0.441	11	0.997
4–5	M	2.979	0.725	29	0.805	3.090	0.888	29	0.922	2.869	0.708	29	0.685

**Fig. 4:**
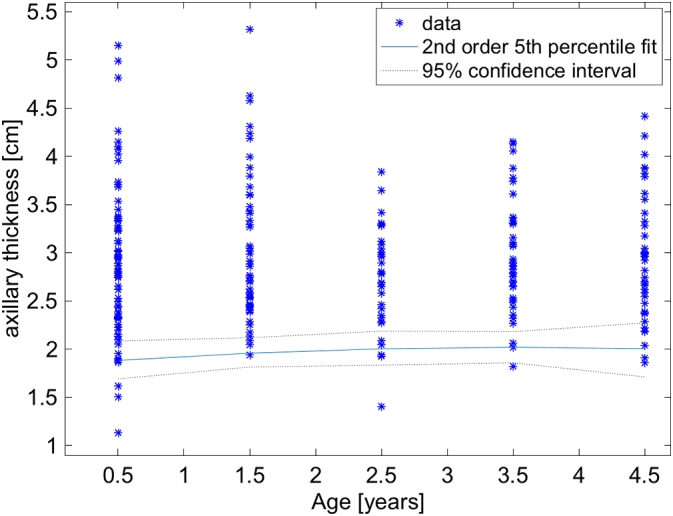
Plot of the distributions of measurements in each of the 5 age cohorts. The 5th percentile is represented by the nearly horizontal blue line with its 95% confidence intervals represented by the dotted black lines. Because the 5th percentile thresholds were not exactly the same and some of the subpopulation graphs shown below have a pronounced curvature for these thresholds, we chose to fit the thresholds with a second order polynomial, i.e. parabola. This did not affect the lowest threshold which is just above 1.5 cm in the 0–1 year old cohort. (For interpretation of the references to color in this figure legend, the reader is referred to the web version of this article.)

**Fig. 5:**
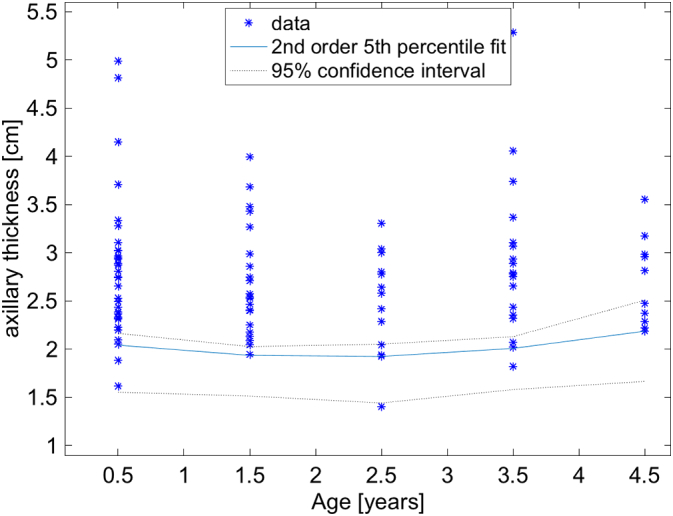
Plot of the distribution of measurements for the girls in the 5 age cohorts. Note that the 5th percentile fit is curved as a function of age. Because of this we fitted the thresholds with a second order polynomial, i.e. a parabola, rather than a straight line as described above. However, a 1.5 cm threshold would still appear appropriate. The blue curve represents the 5th percentile and the dotted black lines are the 95% confidence intervals of the 5th percentile. (For interpretation of the references to color in this figure legend, the reader is referred to the web version of this article.)

**Fig. 6:**
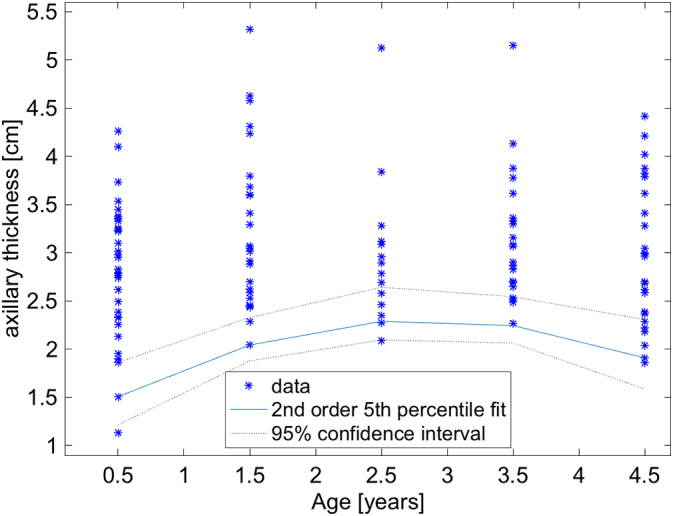
Plot of the distribution of measurements for the boys in the 5 age cohorts. The curvature of the 5th percentile is opposite that for girls, and the curvature is more than for the girls. A second order parabolic fit was again applied to the age thresholds. In this circumstance, a more aggressive threshold may be required in the 0–1 year cohort. The blue curve represents the 5th percentile and the dotted black lines are the 95% confidence intervals of the 5th percentile. (For interpretation of the references to color in this figure legend, the reader is referred to the web version of this article.)

**Fig. 7:**
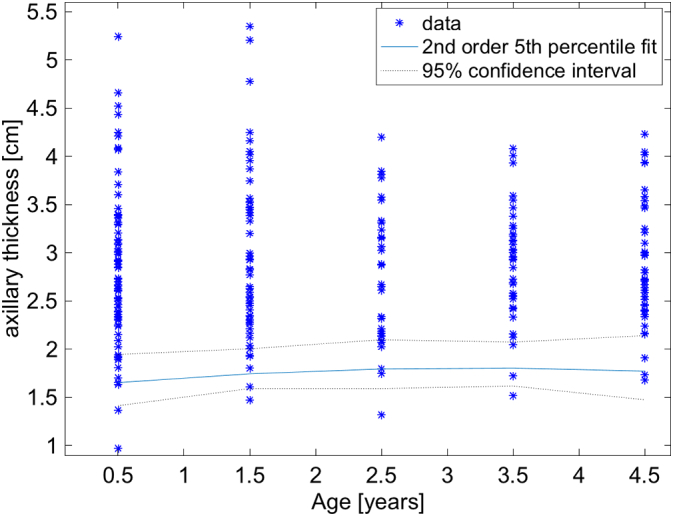
Plot of the distributions of axillary thickness for the left axillae for the entire population. The threshold appears very similar to those of the entire population (Fig. 4). A second order parabolic fit was applied to the thresholds. The blue curve represents the 5th percentile and the dotted black lines are the 95% confidence intervals of the 5th percentile. (For interpretation of the references to color in this figure legend, the reader is referred to the web version of this article.)

**Fig. 8:**
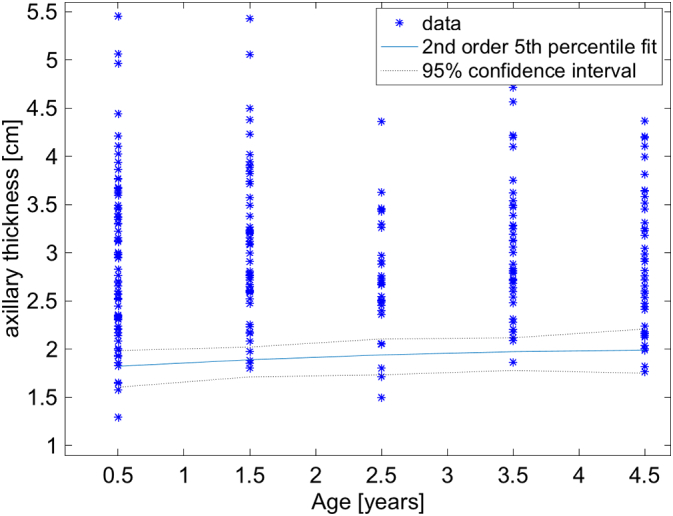
Plot of the distributions of axillary thickness for the right axillae for the entire population. The threshold appears very similar to those of the entire population (Fig. 4). Again, a second order parabolic fit was applied to the thresholds. The blue curve represents the 5th percentile and the dotted black lines are the 95% confidence intervals of the 5th percentile. Even though there is a definite trend for increased thickness of the left axillae over the right (Fig. 3), the 5th percentile thresholds are nearly identical. (For interpretation of the references to color in this figure legend, the reader is referred to the web version of this article.)

**Fig. 9:**
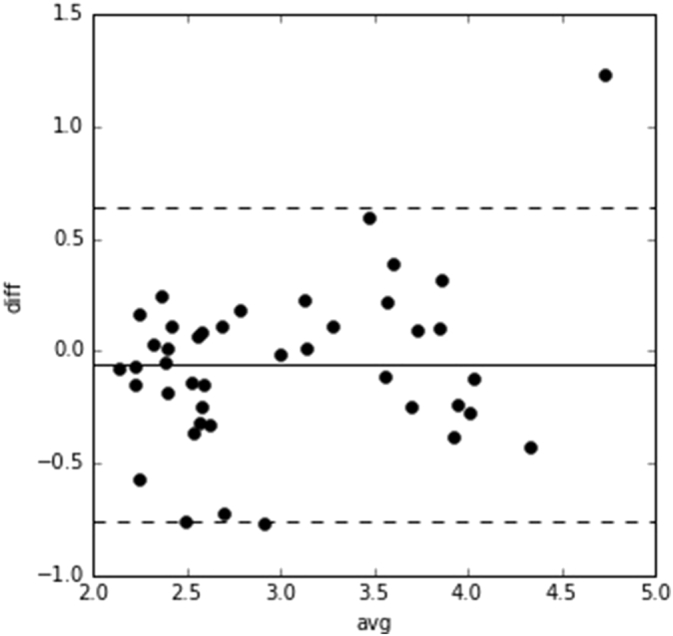
Bland-Altman plot of matched measurements in 41 randomly selected cases. There is no obvious bias is demonstrated, and the 95% confidence limits, dotted lines, include zero, i.e. no difference between the observers. diff = difference measured in centimeters. avg = average measured in centimeters.

Fifth percentile plots show the distributions of the lower bound thresholds for the population as a whole, for boys, for girls, for left axillary, and for right axillary measurements (Fig. 4, Fig. 5, Fig. 6, Fig. 7, Fig. 8). A Bland-Altman plot of the mean measurements in 41 cases measured separately by AWK and JMR showed no trends between the observers with the zero difference line well within the 95% confidence intervals of the measurements (Fig. 9).

## 4 Discussion

Childhood pneumonia is a devastating disease in the third world contributing up to 3 million deaths per year [20]. The seriousness of this disease increases when children are malnourished.

We have undertaken a study to establish axillary thickness distributions and thickness thresholds in non-malnourished children. The results are intended to inform the development of an ultrasound tool that can simultaneously identify nutritional state and lung parenchymal abnormalities. This would be similar to the standard measurements presently used to assess nutritional status with the added advantage of assessing both pulmonary abnormalities and nutritional status without the need for additional maneuvers [15,16].

However, in order to use ultrasound axillary thickness measurements to assess childhood nutrition, one must first know the normal axillary thickness measurement distributions and then determine lower bounds of these distributions. To do this, we produced 5th percentile plots of the entire population as well as distributions of the boys, girls, and left and right sides. In addition, we showed how the means of the population varied from side to side and over the cohort.

Several interesting properties are worth noting. The 5th percentiles for all age groups are remarkably similar (Fig. 4). This is surprising, but makes it simple to estimate if a child is malnourished or not, independent of age. The lower bounds of the 95% confidence intervals of all ages is >1.5 cm. This 1.5 cm threshold and age independence appears to hold for left and right sides as well even though it appears that the mean values of the axillary thickness is larger on the left than the right side (Fig. 3). Therefore, a measurement of either axilla of <1.5 cm would suggest that a child is malnourished. Unfortunately, there appears to be an age dependence for the thresholds stratified by sex. For girls, the 95% lower bound is very close to 1.5 cm in the 2–3 year cohort, and it is a minimum. For boys, the threshold goes below 1.5 cm and approaches 1.1 cm in the 0–1 year cohort. So given this, a 1.5 cm would be a very good greatest lower bound for axillary thickness, except perhaps for boys between 0 and 1 years old where 1.1 cm may need to be used. These thresholds would need to be confirmed with future studies.

Finally, a Bland-Altman analysis of a small random set of measurements made by two observers (AWK and JMR) showed no obvious bias for the equivalent width estimates of axillary thickness used in this analysis. This implies that the equivalent width measurements were generally reproducible and are consistent for their axillary thickness measurements.

There is at least one major limitation with this study. All of these children were considered representative of a nutritionally normal population between the ages 0–5 years old. As mentioned, obtaining such a population is difficult as normal, healthy children generally do not have CT scans. We therefore had to study children with non-serious anomalies, acute illnesses, acute trauma, or acutely presenting masses that had not been diagnosed or treated. Determinations of which cases would or would not be included in the cohort was based the judgment of the investigator after reading the medical record and looking at the indications for the CT scan. These analyses were, therefore, somewhat subjective, but we believe we made a good faith effort in selecting our population. Further, these children largely came from the midwestern and eastern United States. Thus, these thresholds may differ in other parts of the world. However, the remarkably uniform measurement thresholds for detecting malnutrition suggest that these measurements may have a certain robustness. Again, these results will have to be confirmed.

Finally, we used the equivalent width measurement to the estimate of the axillary thickness. We needed a way to compensate for the curved, irregular axillary boundaries produced by the lungs and skin. Other alternatives such as several random linear measurements could have been employed. However, they would ultimately be summarized by a mean ± a standard deviation for each measurement, and we obtained similar statistics over our population with equivalent width. As shown, equivalent width produced an unbiased estimate between two observers (Fig. 9), and could be reasonably used as a means of estimating average axillary thicknesses in our population.

## 5 Conclusion

Axillary skin thickness measurements were made from CT scans of the chest in a cohort of presumably normally nourished children from ages 0 to 5 years. A threshold of 1.5 cm appears to be appropriate for all ages except for boys between 0 and 1 years of age where, based on 5th percentile analysis, a threshold of 1.1 cm may be more appropriate.

The authors have no disclosures.
